# Prospective study of serum B vitamins levels and oesophageal and gastric cancers in China

**DOI:** 10.1038/srep35281

**Published:** 2016-10-17

**Authors:** Jiansong Ren, Gwen Murphy, Jinhu Fan, Sanford M. Dawsey, Philip R. Taylor, Jacob Selhub, Youlin Qiao, Christian C. Abnet

**Affiliations:** 1 Program Office for Cancer Screening in Urban China, National Cancer Center/Cancer Hospital, Chinese Academy of Medical Sciences and Peking Union Medical College, Beijing, China; 2 Division of Cancer Epidemiology and Genetics, National Cancer Institute, National Institutes of Health, Bethesda, Maryland, USA; 3 Department of Epidemiology, National Cancer Center/Cancer Hospital, Chinese Academy of Medical Sciences and Peking Union Medical College, Beijing, China; 4 Jean Mayer U.S. Department of Agriculture Human Nutrition Research Center on Aging at Tufts University, Boston, Massachusetts, USA

## Abstract

B vitamins play an essential role in DNA synthesis and methylation, and may protect against oesophageal and gastric cancers. In this case-cohort study, subjects were enrolled from the General Population Nutrition Intervention Trial in Linxian, China. Subjects included 498 oesophageal squamous cell carcinomas (OSCCs), 255 gastric cardia adenocarcinomas (GCAs), and an age- and sex-matched sub-cohort of 947 individuals. Baseline serum riboflavin, pyridoxal phosphate (PLP), folate, vitamin B12, and flavin mononucleotide (FMN) were measured for all subjects. We estimated the associations with Cox proportional hazard models, with adjustment for potential confounders. Compared to those in the lowest quartile of serum riboflavin, those in the highest had a 44% lower risk of OSCC (HR: 0.56, 95% CI: 0.41 to 0.75). Serum vitamin B12 as a continuous variable was observed to be significantly inversely associated with OSCC (HR: 0.95, 95% CI: 0.89 to 1.01, *P* for score test = 0.041). Higher serum FMN levels were significantly associated with increased risk of OSCC (HR: 1.08, 95% CI: 1.01 to 1.16) and GCA (HR: 1.09, 95% CI: 1.00 to 1.20). Our study prompted that B vitamins have the potential role as chemopreventive agents for upper gastrointestinal cancers.

B vitamins, including riboflavin (vitamin B2), pyridoxal phosphate (PLP or vitamin B6), folate (vitamin B9), and vitamin B12, play an important role in DNA synthesis and methylation acting as coenzymes in one-carbon metabolism^1^. Deficiencies in B vitamins may increase the probability of disruption of this metabolic pathway and result in aberrations in DNA methylation, imbalance in DNA precursors, and deficiency in DNA repair, each of which can contribute to carcinogenesis^2^. Dietary intake of these B vitamins may help to decrease cancer risk because of their role in maintaining DNA integrity.

Until now, most studies of B vitamins and cancer prevention have focused on dietary intake estimates and risk of breast, prostate and colorectal cancers^3^, but serum B vitamin concentrations have previously also been measured mainly in studies of lung and colorectal cancers^1^,^4^. Only a few studies have explored the association between the intake of B vitamins and risk of oesophageal or gastric cancer^5^,^6^,^7^. In an intervention trial conducted in a northern county in China (Cixian), riboflavin-fortified salt was found to reduce the incidence of oesophageal squamous cell carcinoma (OSCC)^8^. Low riboflavin intake has been noted in several areas of the world with elevated oesophageal cancer incidence, including the high-risk areas of north central China, where the majority of the population also shows biochemical evidence of low riboflavin status. In a case-control study, riboflavin intake was observed to be inversely, but non-significantly, associated with the risk of adenocarcinomas of oesophagus and gastric cardia combined^9^. However, a multicenter population-based case-control study in the United States found that the participants with high riboflavin intake had a non-significantly increased risk for OSCC or gastric cardia adenocarcinoma (GCA)^10^.

Oesophageal and stomach cancers remain a significant cause of morbidity and mortality worldwide^11^. Linxian, a rural county in north central China, has among the highest rates of oesophageal and gastric cardia cancers in the world, and its inhabitants have chronic nutritional inadequacies, including low intake and serum levels of multiple B vitamins. When the General Population Nutrition Intervention Trial (NIT) cohort was enrolled, 97% of Linxian residents had biochemical ariboflavinosis (defined as erythrocyte glutathione reductase activation coefficients equal to or greater than 1.30)^12^, and compared with low risk for oesophageal cancer, Linxian had significantly lower riboflavin status, consistent with local food consumption surveys^13^.

In this study we aimed to directly test the association between baseline serum concentrations of B vitamins and subsequent risk of OSCC and GCA using a case-cohort study within the NIT Trial in Linxian, China. All subjects included in our study were part of this randomized clinical trial that included supplementation of half of the participants with riboflavin and niacin and we have accounted for this intervention using adjustments and stratifications to fully explore the associations.

## Methods

### Study cohort

Subjects of this study were selected from the participants of the Linxian General Population NIT trial. We have previously given a detailed description of the design, conduct and results of this trial and its extended follow-up^14^,^15^,^16^. In brief, the participants consisted of 29,584 healthy adults aged 40–69 years at enrollment who resided in one of four Linxian communes. In the spring of 1985, each participant was interviewed, given a brief physical examination, and had 10 ml of blood drawn. After collection, serum specimens were separated, aliquoted, and stored frozen at −80 °C for future analysis. The intervention began in March 1986 and continued through May 1991, lasting 5.25 years. Under a partial factorial design, participants were randomly assigned to receive a daily vitamin-mineral combination or a placebo in the form of individual oral tablets. Four vitamin-mineral combinations were tested: Factor A (5,000 IU of vitamin A and 22.5 mg of zinc oxide); Factor B (3.2 mg of riboflavin and 40 mg of niacin); Factor C (120 mg of ascorbic acid and 30 μg of molybdenum); and Factor D (50 μg of yeast selenium, 15 mg of β-carotene, and 30 mg of α-tocopherol). These doses ranged from one to two times the U.S. Recommended Daily Allowances (RDAs). Throughout the trial period, local healthcare workers recorded cancer incidence and mortality data at monthly intervals, and periodic surveys were conducted to verify the completeness and accuracy of follow-up information. Pathology slides and/or X rays were available for 85% of the cancer cases in this study, and these were reviewed and the case status confirmed by a panel of American and Chinese experts. Outcomes for the current analysis were based on the intervention trial period from March 1986 through May 1991. Less than 1% of the participants were lost to follow-up during this time. The study was conducted under the auspices of the Institutional Review Boards of the National Cancer Center/Cancer Hospital, Chinese Academy of Medical Sciences and the US National Cancer Institute.

### Selection of study participants for case-cohort analysis

During the 5.25 years of the nutritional intervention, 640 OSCCs and 435 GCAs were identified from all participants in the General Population NIT trial. GCAs were defined as the tumors centered in the most proximal 3 cm of the stomach.

Using a stratified case-cohort design^17^,^18^, 498 OSCCs and 255 GCAs were randomly selected from the identified cases for this analysis, following a series of simulations and power calculations to determine study size. A comparison group (sub-cohort) was also generated from a stratified random sample of all trial participants (n = 947, including cases and non-cases). Six strata were defined by sex and age categories (as of the beginning of the trial: <50 years; 50–60 years; and >60 years). The lowest within-stratum ratios of control subjects to case subjects for incident OSCCs or GCAs were 1.4 and 2.7, respectively.

### Serological assays

In 1996, aliquots of the baseline sera from all consenting participants were transferred on dry ice to the National Cancer Institute repository. Samples selected for this study were thawed, aliquoted, and shipped on dry ice to the analytical laboratories (USDA Jean Mayer Human Nutrition Research Center on Aging at Tufts University, Boston, Massachusetts, USA). Serum riboflavin, PLP, folate, vitamin B12 and flavin mononucleotide (FMN) concentrations were determined in a single run using a modified high-performance liquid chromatography method^19^. Each batch contained adjacent blinded case and sub-cohort samples, as well as six blinded quality control samples derived from a pool of serum from Linxian residents. The overall coefficients of variation for riboflavin, PLP, folate, vitamin B12, and FMN in the 153 QC samples were 24%, 37%, 17%, 17% and 33%, respectively.

### Statistical Analysis

Quantile (25^th^, 50^th^ and 75^th^ percentile) values for the serum levels of these exposures were calculated for the entire trial cohort using the sampling weights. Correlation coefficients among these exposures were calculated using Pearson parametric methods. Follow-up time was measured from the start of the intervention (March 1986) to any cancer diagnosis, loss to follow-up, death, or the end of the intervention (May 1991), whichever occurred first. Cox proportional hazards models were used to estimate adjusted hazard ratios (HRs) and 95% confidence intervals (95% CIs) after excluding serum analytes outliers (defined as larger than Mean + 4SD). All Cox models were adjusted for age, sex, cigarette smoking, alcohol drinking, BMI, and Factor B treatment group assignment (except stratified models as noted below). Models were fit using the case-cohort Cox models in Epicure Software (Hirosoft, Seattle, Washington, USA) and *P* values for trend were derived from score tests. We examined the proportional hazards assumption graphically and used lag analyses that excluded cases diagnosed in the first two years and found no evidence for non-proportionality.

In the analyses of the relationship between these serum B vitamins and OSCC or GCA, a number of alternative metrics were used. Riboflavin was supplemented in half of the participants as an ingredient in Factor B (3.2 mg of riboflavin and 40 mg of niacin), so associations with OSCC and GCA were adjusted for Factor B. We also estimated risks separately for those who were, or were not, assigned to a factor B supplementation group.

### Ethics

This study was approved by the Institutional Review Boards of the US National Cancer Institute and the National Cancer Center/Cancer Hospital, Chinese Academy of Medical Sciences. This study was carried out in accordance with the approved guidelines, and all participants provided written informed consent.

## Results

Table 1 shows demographic characteristics and data on selected potential confounders in cancer cases and sub-cohort members. Among the participants, 54% of the sub-cohort participants were males, compared with 48% of OSCC and 65% of GCA cases. Mean ages, alcohol drinking percentages, and mean BMIs were similar among all subgroups compared to the full underlying NIT cohort. GCA cases had a higher prevalence of cigarette smoking (46%) than the sub-cohort (38%) and OSCC (38%) groups.

Table 2 presents the distributions for each analyte in the sub-cohort weighted by the sampling fraction, which provides appropriate estimates for the distribution in the full NIT cohort rather than just in the subjects who did not develop cancer during the follow-up period.

The Pearson correlation coefficients for the associations between serum riboflavin, PLP, folate, B12, and FMN are shown in Table 3. Serum concentrations of these B vitamins were weakly correlated with each other (*r *= 0.02–0.10) among the sub-cohort participants, except for a relatively strong correlation between riboflavin and FMN (*r* = 0.24).

When modeled as a continuous variable, serum riboflavin was significantly inversely associated with the risk of OSCC (HR: 0.94; 95% CI: 0.90 to 0.98) (Table 4). In the analysis using serum quartiles, subjects in the highest quartile of serum riboflavin distribution, compared to those in the lowest quartile, had a significantly lower risk of OSCC (HR: 0.56; 95% CI: 0.41 to 0.75) and the test for trend was significant (*P* < 0.001). After stratifying by assignment to Factor B, a stronger association was seen for the participants who did not receive Factor B (fourth *vs*. first quartile HR: 0.51; 95% CI, 0.33 to 0.78) than for those who did receive this supplementation (fourth *vs.* first quartile HR, 0.60; 95% CI, 0.39 to 0.93). No significant association was observed overall for serum riboflavin and GCA (continuous model HR: 0.97; 95% CI: 0.92 to 1.03; *P* = 0.25), and the decreasing trend in quartile HRs was not significant when the analysis was limited to the participants not assigned to Factor B supplementation (*P* for trend = 0.055).

Slight but significant associations were seen between participants with higher serum folate levels and GCA (continuous model HR: 1.08; 95% CI: 1.00 to 1.17; *P* = 0.035 per 0.84 nmol/L increase in concentration), but this association was not significant when stratified by status of Factor B supplements. No significant association was seen between serum folate level and OSCC (continuous model HR: 1.05; 95% CI: 0.98 to 1.12; *P* = 0.079 per 0.84 nmol/L increase in concentration), although a significant association was observed among Factor B recipients (continuous model HR: 1.10; 95% CI: 1.01 to 1.20; *P* = 0.0070).

No significant associations were observed between PLP and OSCC or GCA in continuous models. However, subjects in the second and third quartiles of serum PLP had an increased risk of OSCC (HR: 1.40, 95% CI: 1.04 to 1.89 and HR: 1.47, 95% CI: 1.08 to 1.98, respectively).

Serum vitamin B12 as a continuous variable was inversely associated with OSCC (HR: 0.95, 95% CI: 0.89 to 1.01; *P* = 0.041), however, no association was seen when participants were stratified by Factor B supplement status or when the analyses were performed as quartiles. There was no evidence of an association for serum B12 with GCA in any of our analyses.

A non-significant increased risk of OSCC was seen among subjects with higher serum FMN levels (continuous model HR: 1.05; 95% CI: 0.98 to 1.12; *P* = 0.080: per 4.71 nmol/L increase in concentration). Significant association was seen among the participants who received the Factor B supplement (continuous model HR: 1.12; 95% CI: 1.02 to 1.23; *P* = 0.0041). In the quartile analysis, no significant association was observed for FMN and OSCC. A similar non-significant increase in risk was seen for higher serum FMN levels and GCA (continuous model HR: 1.06; 95% CI: 0.97 to 1.15; *P* = 0.15). In light of the correlation between riboflavin and FMN, we also fit models that included both analytes simultaneously. Compared with the single analyte analyses above, both the association between riboflavin and OSCC, and that between FMN and OSCC appeared stronger (Table 5).

**Figure 1:**
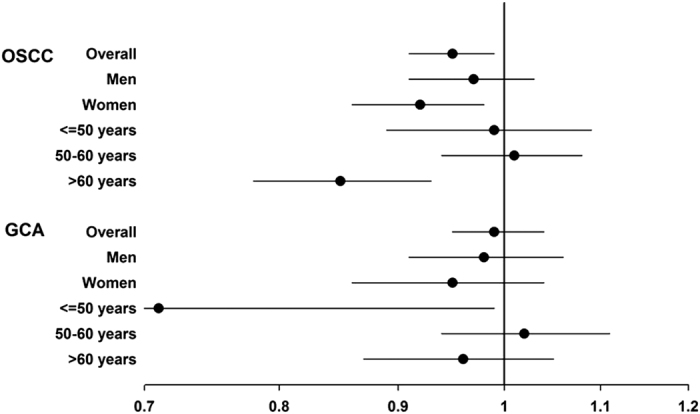
Plot of HRs and 95% CIs for the association between serum riboflavin and risk of OSCC and GCA overall and by sex or age in the Linxian Nutrition intervention Trial cohort (HRs and 95% CIs were calculated using model adjusted for age, sex, cigarette smoking, alcohol drinking, BMI, and Factor B, as appropriate).

We further explored the association between riboflavin and OSCC after stratifying by sex and age (Fig. 1). The overall inverse associations between serum riboflavin and OSCC were stronger in women (HR: 0.92, 95% CI: 0.86 to 0.98; *P *= 0.009) and in participants older than 60 years (HR: 0.85, 95% CI: 0.78 to 0.93; *P *< 0.001).

The association between riboflavin and GCA was null overall but a significant inverse association was seen among participants <=50 years old (HR: 0.71, 95% CI: 0.51 to 0.99; *P* = 0.044; and Fig. 1).

## Discussion

This is the first study to evaluate the relationship between serum riboflavin level and risk of oesophageal or gastric cancer. In this large prospective study nested in a clinical trial cohort we found that higher serum riboflavin concentrations were significantly associated with reduced risk of OSCC. Those in the highest quartile of serum riboflavin had a 44% lower risk of OSCC compared with those in the lowest quartile. This inverse association was stronger for women and those older than 60 years. We also analyzed this observational data separately in persons who did and did not receive riboflavin supplementation during the trial, and a larger effect was seen when we restricted our analysis to subjects without riboflavin supplements. Because serum riboflavin levels were affected by supplementation during the trial period^15^, the association derived from the participants without riboflavin supplement may be the better estimate of the true association. We previously reported that Factor B supplementation did not reduce the risk of oesophageal or gastric cardia cancer in this study, although a reduced risk for OSCC incidence was suggested for results from both the trial period (RR = 0.86, 95% CI = 0.74–1.01)^15^ as well as the 15.25-year combined trial plus post-trial period (HR = 0.93, 95% CI = 0.84–1.02)^16^. This apparent discrepancy between the trial results and the observational analysis may reflect the limitations of starting supplementation in adults exposed to a life time of nutritional deficiency and using relatively modest doses in a low-status population.

B vitamins are provided in the diet from many foods, such as whole-grain cereals, rice, nuts, milk, eggs, meat, fish, fruits, and leafy green vegetables. B vitamins maintain and increase the metabolic rate and promote growth and cell division. PLP, folate, and vitamin B12 have a number of interrelated biological roles that make them potentially important agents in cancer. Perhaps most importantly, they function as coenzymes in the synthesis of purines and thymidylate for DNA synthesis. When B vitamin levels are insufficient, the initiation of cancer is facilitated by reduction of thymidylate synthesis, resulting in increased incorporation of uracil in DNA and consequent chromosome breaks, disruption of DNA repair, and neoplastic transformation^20^,^21^. The increased chromosome breakage associated with low levels of folate, vitamin B12, or elevated status of homocysteine has been previously demonstrated^22^. Folate and vitamin B12 are also critical in methylation reactions in the human body.

The residents of Linxian, and NIT trial participants have consistently been observed to have low levels of riboflavin in blood, urine, and diet, including 77% to 97% with riboflavin-deficient levels^12^,^13^,^23^. The low protein intake in this population may explain the relatively low serum riboflavin concentrations. The concentration of serum riboflavin we observed in the Linxian cohort (median: 13.5 pmol/ml; inter-quartile range (IQR): 9.9–18.3 pmol/ml) was much lower than that reported in the European Prospective Investigation into Cancer and Nutrition (EPIC) study (median 19.7 pmol/ml)^1^.

In addition to the riboflavinized salt trial mentioned above^23^, a double-blind placebo-controlled intervention trial in Huixian, China found that subjects who were given retinol, riboflavin (200 mg, once a week), and zinc for one year did not show a different prevalence of precancerous lesions of oesophagus than the placebo group. However, those individuals who had a larger increase in their riboflavin blood level during the intervention were more likely to have a histologically normal oesophagus at the end of the trial^24^.

We did not find any convincing evidence for a relation between serum riboflavin and GCA risk, although quartile analysis showed a nearly significant trend between higher serum riboflavin and lower risk of GCA, and stratified analyses indicated a protective association in younger person.

There is limited but inconclusive epidemiological evidence that high dietary folate intake could reduce the risk of esophageal cancer. Our study did not show significant association between serum folate and OSCC or GCA. Similarly, a study in Australia showed that high intake of folate was not associated with the risk of OSCC^25^. A study in Urguay, however, found a decreased risk of esophageal cancer with a high dietary folate intake, but the cases of esophageal cancer were not divided by histology^26^.

Serum PLP was not found significantly associated with OSCC or GCA in our study, this is consistent in part with the findings of a study from Australia, which also reported nonsignificant association between PLP intake and the risk of OSCC^25^.

In this study, serum vitamin B12 was inversely associated with OSCC, but this association was limited to the the analysis where serum vitamin B12 as a continuous variable. Comparablly, in a study conducted in the United States of America, no association was found between intake of dietary vitamin B12 and OSCC or GCA^6^.

FMN is a phosphoric ester of riboflavin that constitutes the cofactor of various flavoproteins. In this study FMN was correlated with riboflavin, which is consistent with previous studies^27^. Stronger associations between riboflavin and OSCC and FMN and OSCC were observed when riboflavin and FMN were jointly modeled, compared with the single analyte analyses. We suspect that because both riboflavin and FMN were associated with OSCC, but with opposite effects, a joint model better estimates the net effect for each exposure and thus the associations strengthened.

Our study has a number of strengths, including its prospective study design, the large number of cancer cases, the availability of data on potential confounders, state-of-the-art measurement methods for serum B vitamin concentrations, and the virtually complete follow-up of all study participants. However, we also note several limitations. High coefficients of variation for the measured serum nutrients were observed in some quality control samples which likely reduced our power. There is always a concern that pre-clinical disease could lead to alteration in serum measurements, thus creating a misleading association, although our lag-analysis suggests this was not a significant problem in the current study. And finally, the generalizability of our study may be limited due to the specifics of the population under study.

In summary, in this large prospective study, we found that serum concentrations of several B vitamins and related metabolites were significantly associated with risk of OSCC or GCA. Particularly, higher serum riboflavin was associated with significantly lower risk of OSCC. Riboflavin should be further investigated for its potential role as a chemopreventive agent for upper gastrointestinal cancers.

## Additional Information

**How to cite this article**: Ren, J. *et al*. Prospective study of serum B vitamins levels and oesophageal and gastric cancers in China. *Sci. Rep.* **6**, 35281; doi: 10.1038/srep35281 (2016).

## Figures and Tables

**Table 1:** Study characteristics of cases and the sub-cohort from the Linxian General Population Nutrition Intervention Trial cohort, overall and by sex.

	Sub-cohort	OSCC	GCA
Number, total N (%)	947	498	255
Men	515 (54%)	241 (48%)	167 (65%)
Women	432 (46%)	257 (52%)	88 (35%)
Age at baseline, mean (SD)	58 (7.7)	57 (7.8)	58 (6.4)
Men	59 (6.8)	58 (7.0)	58 (6.1)
Women	56 (8.4)	55 (8.3)	56 (6.7)
Cigarette smoking, N (%) yes^a^	364 (38%)	190 (38%)	118 (46%)
Men	362 (70%)	190 (79%)	118 (71%)
Women	2 (<1%)	0 (0%)	0 (0%)
Alcohol drinking, N (%) yes^b^	200 (21%)	112 (23%)	58 (23%)
Men	172 (33%)	95 (39%)	51 (31%)
Women	28 (6%)	17 (7%)	7 (8%)
BMI (kg/m^2^), mean (SD)	21.8 (2.6)	21.4 (2.4)	21.6 (2.5)
Men	21.5 (2.1)	21.6 (2.1)	21.3 (2.0)
Women	22.1 (3.1)	21.3 (2.6)	22.1 (3.2)

^a^Daily cigarette consumption among men was generally low and few subjects had quit smoking, so smoking is presented as never smoking versus ever smoking.

^b^Alcohol consumption was minimal at the time of the baseline interview (1985), so alcohol drinking was categorized as any consumption in the previous 12 months versus none.

**Table 2:** Quartiles for serum B vitamin distributions in the Linxian General Population Nutrition Intervention Trial cohort.

Exposures	Quantile^a^		
	25^th^	50^th^	75^th^
Riboflavin (nmol/L)	9.9	13.5	18.3
PLP (nmol/L)	19.2	26.5	36.3
Folate (nmol/L)	2.8	3.5	4.5
B12 (pmol/L)	111.5	156.6	213.6
FMN (nmol/L)	36.1	40.4	45.5

^a^Weighted by the entire NIT cohort.

**Table 3:** Pearson correlation coefficients^a^ between serum B vitamin concentrations in the sub-cohort subjects.

	PLP	Folate	B12	FMN
Riboflavin	0.09567	0.09799	0.10076	0.24279
	*P* = 0.0033	*P* = 0.0026	*P* = 0.0019	*P* < 0.0001
PLP		0.08823	0.04934	0.02628
		*P* = 0.0066	*P* = 0.1292	*P* = 0.4202
Folate			0.01846	0.08398
			*P* = 0.5707	*P* = 0.0099
B12				0.02240
				*P* = 0.4921

^a^Weighted by the entire NIT cohort.

**Table 4:** HRs and 95% CIs^a^ for the association between serum B vitamin concentrations and risk of OSCC and GCA.

		HR	Continuous^b^		Quartile							
			95% CI	*P* Value	Q1	Q2		Q3		Q4		
					Ref	HR	95% CI	HR	95% CI	HR	95% CI	*P*_trend_^c^
Riboflavin	**OSCC**											
	Overall	0.94	0.90 to 0.98	0.0024	1.00	0.70	0.52 to 0.94	0.83	0.62 to 1.10	0.56	0.41 to 0.75	<0.001
	Factor B recipients	0.95	0.88 to 1.02	0.079	1.00	0.67	0.43 to 1.03	0.77	0.50 to 1.17	0.60	0.39 to 0.93	0.039
	Factor B non-recipients	0.93	0.88 to 0.99	0.011	1.00	0.71	0.47 to 1.08	0.86	0.58 to 1.29	0.51	0.33 to 0.78	0.0096
	**GCA**											
	Overall	0.97	0.92 to 1.03	0.25	1.00	0.87	0.60 to 1.27	0.86	0.59 to 1.25	0.69	0.47 to 1.02	0.070
	Factor B recipients	1.00	0.92 to 1.09	>0.50	1.00	0.77	0.44 to 1.36	1.01	0.59 to 1.73	0.81	0.47 to 1.40	>0.50
	Factor B non-recipients	0.94	0.87 to 1.03	0.14	1.00	0.96	0.57 to 1.62	0.77	0.44 to 1.33	0.59	0.33 to 1.05	0.055
PLP	**OSCC**											
	Overall	1.02	0.96 to 1.09	>0.50	1.00	1.40	1.04 to 1.89	1.47	1.08 to 1.98	1.19	0.86 to 1.66	0.15
	Factor B recipients	1.01	0.92 to 1.10	>0.50	1.00	1.31	0.84 to 2.04	1.37	0.88 to 2.12	1.20	0.74 to 1.92	0.32
	Factor B non-recipients	1.03	0.93 to 1.12	>0.50	1.00	1.43	0.95 to 2.16	1.51	0.97 to 2.34	1.13	0.71 to 1.81	0.44
	**GCA**											
	Overall	1.05	0.96 to 1.14	0.23	1.00	1.42	0.98 to 2.05	1.28	0.86 to 1.89	1.16	0.75 to 1.78	0.43
	Factor B recipients	1.01	0.89 to 1.13	>0.50	1.00	1.39	0.82 to 2.36	1.16	0.67 to 2.01	1.05	0.57 to 1.93	>0.50
	Factor B non-recipients	1.10	0.97 to 1.25	0.093	1.00	1.37	0.81 to 2.31	1.32	0.74 to 2.36	1.26	0.67 to 2.35	0.44
Folate	**OSCC**											
	Overall	1.05	0.98 to 1.12	0.079	1.00	0.77	0.57 to 1.05	0.90	0.66 to 1.21	1.06	0.79 to 1.43	>0.50
	Factor B recipients	1.10	1.01 to 1.20	0.0070	1.00	0.73	0.47 to 1.16	0.70	0.44 to 1.10	1.18	0.77 to 1.81	0.46
	Factor B non-recipients	0.99	0.90 to 1.09	>0.50	1.00	0.79	0.52 to 1.21	1.07	0.70 to 1.63	0.92	0.60 to 1.43	>0.50
	**GCA**											
	Overall	1.08	1.00 to 1.17	0.035	1.00	0.88	0.59 to 1.30	1.08	0.74 to 1.59	1.28	0.87 to 1.89	0.15
	Factor B recipients	1.07	0.96 to 1.20	0.18	1.00	1.31	0.75 to 2.29	1.07	0.60 to 1.91	1.46	0.82 to 2.59	0.32
	Factor B non-recipients	1.09	0.97 to 1.22	0.12	1.00	0.55	0.30 to 0.99	1.05	0.62 to 1.78	1.10	0.64 to 1.90	0.44
B12	**OSCC**											
	Overall	0.95	0.89 to 1.01	0.041^d^	1.00	0.79	0.59 to 1.05	0.84	0.62 to 1.12	0.84	0.62 to 1.13	0.30
	Factor B recipients	0.96	0.88 to 1.05	0.29	1.00	0.88	0.58 to 1.35	0.98	0.63 to 1.52	0.88	0.56 to 1.38	>0.50
	Factor B non-recipients	0.94	0.85 to 1.03	0.097	1.00	0.73	0.49 to 1.10	0.76	0.51 to 1.14	0.82	0.54 to 1.26	0.36
	**GCA**											
	Overall	0.98	0.90 to 1.06	>0.50	1.00	0.75	0.52 to 1.09	0.75	0.51 to 1.11	0.95	0.65 to 1.39	>0.50
	Factor B recipients	1.00	0.89 to 1.11	>0.50	1.00	0.62	0.36 to 1.06	0.55	0.30 to 0.99	0.98	0.58 to 1.65	>0.50
	Factor B non-recipients	0.95	0.84 to 1.08	0.42	1.00	0.91	0.53 to 1.56	0.97	0.57 to 1.64	0.88	0.49 to 1.58	>0.50
FMN	**OSCC**											
	Overall	1.05	0.98 to 1.12	0.080	1.00	0.93	0.69 to 1.26	0.90	0.66 to 1.22	1.16	0.86 to 1.58	0.40
	Factor B recipients	1.12	1.02 to 1.23	0.0041	1.00	0.93	0.59 to 1.46	0.89	0.56 to 1.41	1.51	0.95 to 2.40	0.088
	Factor B non-recipients	0.99	0.90 to 1.09	>0.50	1.00	0.92	0.61 to 1.40	0.90	0.59 to 1.38	0.91	0.59 to 1.39	>0.50
	**GCA**											
	Overall	1.06	0.97 to 1.15	0.15	1.00	0.72	0.48 to 1.08	1.12	0.77 to 1.64	0.97	0.64 to 1.45	>0.50
	Factor B recipients	1.07	0.94 to 1.21	0.25	1.00	0.62	0.34 to 1.13	1.16	0.68 to 1.98	0.96	0.52 to 1.75	0.48
	Factor B non-recipients	1.03	0.91 to 1.16	>0.50	1.00	0.77	0.43 to 1.37	1.00	0.57 to 1.74	0.89	0.51 to 1.56	>0.50

^a^HRs and 95% CIs were calculated using models adjusted for age, sex, cigarette smoking, alcohol drinking, and BMI. Overall models were also adjusted for receipt of Factor B.

^b^Continuous HRs were scaled to one-half IQR, which was 4.22 nmol/L for riboflavin, 8.51 nmol/L for PLP, 0.84 nmol/L for folate, 51.03 pmol/L for B12, and 4.71 nmol/L for FMN, respectively.

^c^*P* for trend came from a model where quartiles of B vitamins were entered as ordinal variables.

^d^*P* value was not consistent with confidence interval because it was derived from score test.

**Table 5:** Single and joint analyte analysis on the association between Riboflavin and FMN and OSCC and GCA.

Site	Stratification	Model including riboflavin			Model including FMN			Model including (riboflavin + FMN)					
		Riboflavin			FMN			Riboflavin			FMN		
		β	HR^a^	95% CI	β	HR^a^	95% CI	β	HR^a^	95% CI	β	HR^a^	95% CI
OSCC	Overall	−0.06145	0.94	0.90 to 0.98	0.04936	1.05	0.98 to 1.12	−0.07720	0.93	0.88 to 0.97	0.08037	1.08	1.01 to 1.16
	Factor B recipients	−0.05308	0.95	0.88 to 1.02	0.1123	1.12	1.02 to 1.23	−0.07975	0.92	0.86 to 0.99	0.1421	1.15	1.04 to 1.28
	Factor B non-recipients	−0.07031	0.93	0.88 to 0.99	−0.00958	0.99	0.90 to 1.09	−0.07830	0.92	0.87 to 0.99	0.02313	1.02	0.93 to 1.13
GCA	Overall	−0.03049	0.97	0.92 to 1.03	0.05572	1.06	0.97 to 1.15	−0.04251	0.96	0.90 to 1.02	0.08883	1.09	1.00 to 1.20
	Factor B recipients	0.001703	1.00	0.92 to 1.09	0.06574	1.07	0.94 to 1.21	−0.01375	0.99	0.91 to 1.07	0.1027	1.11	0.97 to 1.26
	Factor B non-recipients	−0.05729	0.94	0.87 to 1.03	0.02996	1.03	0.91 to 1.16	−0.06492	0.94	0.86 to 1.02	0.05851	1.06	0.93 to 1.20

^a^HRs and 95% CIs were calculated using models adjusted for age, sex, cigarette smoking, alcohol drinking, and BMI. Overall models were also adjusted for receipt of Factor B. Continuous HRs were scaled to one-half IQR, which was 4.22 nmol/L for riboflavin, and 4.71 nmol/L for FMN, respectively.
